# New insights on tuberculosis transmission dynamics and drug susceptibility profiles among the prison population in Southern Brazil based on whole-genome sequencing

**DOI:** 10.1590/0037-8682-0181-2022

**Published:** 2023-02-20

**Authors:** Lívia Maria Pala Anselmo, Juliana Failde Gallo, Juliana Maira Watanabe Pinhata, Kamila Chagas Peronni, Wilson Araújo da Silva, Patricia de Cássia Ruy, Emilyn Costa Conceição, Anzaan Dippenaar, Robin Mark Warren, Aline Aparecida Monroe, Rosangela Siqueira Oliveira, Valdes Roberto Bollela

**Affiliations:** 1 Universidade de São Paulo, Faculdade de Medicina de Ribeirão Preto, Departamento de Clínica Médica, Ribeirão Preto, SP, Brasil.; 2 Instituto Adolfo Lutz, Núcleo de Tuberculose e Micobacterioses, Centro de Bacteriologia, São Paulo, SP, Brasil.; 3 Instituto para Pesquisa do Câncer, Guarapuava, PR, Brasil.; 4 Universidade de São Paulo, Faculdade de Medicina de Ribeirão Preto, Departamento de Genética, Ribeirão Preto, São Paulo, Brasil.; 5 Universidade de São Paulo, Faculdade de Medicina de Ribeirão Preto, Centro de Medicina Genômica do Hospital das Clínicas, Ribeirão Preto, SP, Brasil.; 6 Department of Science and Innovation - National Research Foundation Centre of Excellence for Biomedical Tuberculosis Research, South African Medical Research Council Centre for Tuberculosis Research, Division of Molecular Biology and Human Genetics, Faculty of Medicine and Health Sciences, Stellenbosch University, Cape Town, South Africa.; 7 Family Medicine and Population Health (FAMPOP), Faculty of Medicine and Health Sciences, University of Antwerp, Antwerp, 2000, Belgium.; 8 Department of Science and Innovation - National Research Foundation Centre of Excellence for Biomedical Tuberculosis Research, South African Medical Research Council Centre for Tuberculosis Research, Division of Molecular Biology and Human Genetics, Faculty of Medicine and Health Sciences, Stellenbosch University, Cape Town, South Africa.; 9 Universidade de São Paulo, Escola de Enfermagem de Ribeirão Preto (EERP-USP), Ribeirão Preto, SP, Brasil.

**Keywords:** Tuberculosis, Prisons, Whole-genome sequencing, Drug-resistant tuberculosis

## Abstract

**Background:**

The rate of tuberculosis (TB) infection among the prison population (PP) in Brazil is 28 times higher than that in the general population, and prison environment favors the spread of TB.

**Objective:**

To describe TB transmission dynamics and drug resistance profiles in PP using whole-genome sequencing (WGS).

**Methods:**

This was a retrospective study of *Mycobacterium tuberculosis* cultivated from people incarcerated in 55 prisons between 2016 and 2019; only one isolate per prisoner was included. Information about movement from one prison to another was tracked. Clinical information was collected, and WGS was performed on isolates obtained at the time of TB diagnosis.

**Results:**

Among 134 prisoners included in the study, we detected 16 clusters with a total of 58 (43%) cases of *M. tuberculosis*. Clusters ranged from two to seven isolates with five or fewer single nucleotide polymorphism (SNP) differences, suggesting a recent transmission. Six (4.4%) isolates were resistant to at least one anti-TB drug. Two of these clustered together and showed resistance to rifampicin, isoniazid, and fluoroquinolones, with 100% concordance between WGS and phenotypic drug-susceptibility testing. Prisoners with clustered isolates had a high amount of movement between prisons (two to eight moves) during the study period.

**Conclusions:**

WGS demonstrated the recent transmission of TB within prisons in Brazil. The high movement among prisoners seems to be related to the transmission of the same *M. tuberculosis* strain within the prison system. Screening for TB before and after the movement of prisoners using rapid molecular tests could play a role in reducing transmission.

## INTRODUCTION

The risk of developing tuberculosis (TB) increases with poverty as well as within vulnerable groups, such as indigenous populations, people living with HIV, prison populations (PPs), and people experiencing homelessness. PP has 28 times more risk of developing active TB than the general population, and this group contributes up to 12% of the newly diagnosed TB cases in Brazil^1^. Globally, prisons have been recognized as institutions with an extremely high TB burden compared to the general population and are known to be reservoirs of persistent multidrug-resistant (MDR) TB^2^. The transmission of TB in a prison is driven by the amount of air shared by the PP, number of people per cell, duration of incarceration, and presence of infected individuals in close contact with the vulnerable imprisoned population. Large reductions in TB transmission were observed when ventilation was improved to meet the WHO recommendations for healthcare settings; interventions after individuals develop symptoms may be too late to control TB spread^3^. International institutions have increasingly recognized the importance of treating the health of the PP as an inseparable component of public health. The growth of the prison population (in London, Poland, France, Germany, Spain, and Italy) threatens public health because it increases the total number of people exposed to TB, facilitating its spread within prisons and, ultimately, to the general public^4^. 

The 2019 Reservoirs of Injustice report by the Yale Global Health Justice Partnership describes how incarceration fuels the spread of TB in Brazil. Black individuals with low socioeconomic status and educational attainment are more likely to be incarcerated and therefore face a higher risk of contracting TB and spreading it to their communities upon release^5^. Walter *et al*. (2021) demonstrated an extraordinarily high risk of acquiring TB in prisons in Central and South America, which creates a health and human rights crisis for the PP and undermines efforts to control TB in this region. Brazil had the highest growth in the absolute number of reported TB diagnoses in prisons between 2015 and 2019, although TB cases declined in the general population^6^.

Universal and systematic genotyping of *Mycobacterium tuberculosis* is fundamental for understanding the molecular epidemiology of TB. Whole-genome sequencing (WGS) can describe genomic variants at a higher resolution and provide comprehensive drug susceptibility profiles^7^ ^-^ ^9^. Using WGS, this study characterized TB transmission dynamics and assessed drug-resistant TB (DR-TB) among prisoners in 55 prisons in a highly populated region of Brazil, using a combination of epidemiology, microbiology, and genomics.

## METHODS

### Study population

*M. tuberculosis* isolates were collected from the PP for diagnostic purposes between 2016 and 2019 in 55 prisons in the northern region of São Paulo, Brazil. All isolates were stored at -70 ºC in the Central Laboratory of *Mycobacteria* (Adolfo Lutz Central Institute) following routine identification and drug susceptibility testing (DST) for first- and second-line anti-TB drugs. A subset of isolates was randomly chosen from the Central Laboratory of *Mycobacteria* to be grown and subjected to WGS.

### Clinical and patient data

Clinical data, complementary test results, and smear microscopy results were obtained from the official databases for the notification of new TB and DR TB diagnosis in Brazil: TB-WEB and SITE-TB (http://sitetb.saude.gov.br/sitetb/login.seam), respectively. The São Paulo State Penitentiary Administration Secretariat provided information about the time when each TB diagnosis was confirmed, including data related to the movement of each prisoner within the system before and after TB confirmation.

### Phenotypic drug-susceptibility tests

*M. tuberculosis* isolates were tested to assess drug susceptibility (DST) to isoniazid (H), rifampicin (R), streptomycin (S), ethambutol (E), fluoroquinolones (Fq), and second-line injectable drugs (SLID): kanamycin (Km), capreomycin (Cm), and amikacin (Am). Phenotypic DST (pDST) was performed using the non-radiometric method in BACTEC MGIT 960 liquid medium (Becton Dickinson Diagnostic Systems, Sparks, MD, USA). First-line DST was performed as described by Adami *et al*. (2017)^10^ and second-line DST was performed following Gallo *et al*. (2017)^11^. 

### Whole-genome sequencing

For WGS, genomic DNA was extracted using the cetyl trimethylammonium bromide (CTAB) technique. Purified genome quality was confirmed by spectrophotometry to have a 260/280 absorbance ratio between 1.8 and 2, and genomic DNA integrity was visualized on agarose gel. Genomic DNA libraries were prepared using a Nextera DNA Sample Prep Kit (Illumina Inc., San Diego, CA, USA) and quantified by fluorometry using a Qubit Fluorometer (Thermo Fisher Scientific, Waltham, USA). A total of 50 ng of DNA from each sample was used for the library preparation. Genomic DNA was fragmented by tagmentation using transposases, followed by library amplification and quantification. In addition to fluorometric quantification, absolute quantification by real-time polymerase chain reaction (PCR) was performed using the KAPA SYBR FAST kit (KAPA Biosystems, Wilmington, USA). Finally, sequencing was performed on an Illumina NextSeq 500 platform (Illumina, San Diego, CA, USA; ILLUMINA SUPPORT CENTER, 2017).

### WGS data analysis

Raw sequencing reads were deposited in the European Nucleotide Archive (project accession number: PRJEB48543). After sequencing, the quality of raw reads was assessed using FastQC v0.11.9, before and after read trimming, using Trimmomatic v0.32 (Bolger *et al*., 2014). The trimmed reads were aligned to a reference genome (*M. tuberculosis* H37Rv, GenBank NC000962.3) using the Burrows-Wheeler Aligner (BWA) v0.7.17 (Li & Durbin, 2009), Novoalign v3.0 (Novocraft Technologies Sdn Bhd, Petaling Jaya, Malaysia), and SMALT v0.7.5^12^. Subsequently, high-confidence genomic variants [Single Nucleotide Polymorphism (SNP) and insertions and deletions (InDels) of 1-10 base pairs] were extracted from the three alignments with SAMTools v1.3 (Li *et al*., 2009) and the Genome Analysis Toolkit (GATK) v3.5^13^. For phylogenetic and cluster analyses, variants in *pe/ppe* genes and regions annotated as ‘insertion sequences and phages’ were excluded, and an allele frequency cutoff of 0.95 was used. 

Sequences of high-confidence variable sites (SNPs only) were used to determine clustering among the study isolates. Genomic transmission clusters were evaluated using a threshold of five or fewer SNPs. Cluster analysis was performed using the R-programming language, with the APE (analyses of phylogenetics and evolution) and ADEGENET (exploratory analysis of genetic and genomic data) packages. In addition, pairwise comparisons were performed only on clustered isolates to determine the absolute variant distance between these isolates. 

Phylogenetic analyses were performed as described by Meiring *et al.* (2020)^14^. A maximum likelihood phylogeny was constructed using IQ-TREE with 1000 bootstrap pseudo-replicates on concatenated sequences containing 33442 high-confidence variable sites. The interactive tree of life (iTOL) v.6^15^ was used to annotate the phylogenetic trees. The final tree included all *M. tuberculosis* isolates and 33 publicly available genomes representative of the different *M. tuberculosis* lineages to provide the structure.

The TB-profiler v.2.3.0^16^was used for drug resistance and lineage prediction. Additionally, RD-Analyzer v1.01^17^ was used to confirm the lineages and SpoTyping-v2.0^18^ was used to predict spoligotyping profiles. The patterns were submitted to the SITVIT2 database (http://www.pasteur-guadeloupe.fr:8081/SITVIT2/description.jsp) for genotyping the *M. tuberculosis* complex. 

### Ethical approval

This project was approved by the Institutional Review Board of Hospital das Clínicas from Ribeirão Preto School of Medicine, University of São Paulo (process number CAAE: 00613218.3.0000.5440), and the Research Ethics Committee of the Secretariat of Penitentiary Administration CEP/SAP Nº065/2018 (process number: 0011042-86.2018.8.26.0496). 

## RESULTS

### Demographic characteristics

Almost all *M. tuberculosis* isolates (131 out of 134) were collected from respiratory specimens (sputum), the majority of which were obtained from male prisoners with an average age of 31 years. Three isolates (3/134) were obtained from women, with an average age of 29 years. Four (4/134) patients had confirmed human immunodeficiency virus (HIV) infection.

### Clinical and molecular epidemiology

All 134 isolates (2.4% of all cases) were obtained from patients who were incarcerated when TB diagnosis was confirmed. A hundred and two (76%) patients with TB were classified as new cases, while 32 (24%) had been previously treated for TB. After treatment was initiated, 127 (94.8%) patients were considered cured, four (3.0%) abandoned and did not complete treatment, and two (1.5%) experienced treatment failure with one leading to death. 

The mean length of incarceration in this group was 40 months (min: 12; max: 96). Out of the 55 different prisons observed, the number of different prisons that each prisoner moved through varied from one to eight (median of three) during the study period. Most prisoners (60.4%) were additionally granted temporary freedom for some time due to an imprisonment regimen change. This change granted temporary freedom for special conditions, such as holidays, and led to a shift in the prisoners´ condition that might change from a closed system (all time in prison) to a semi-open situation when they go out and return to the prison.

### Cluster analysis

**FIGURE 1:**
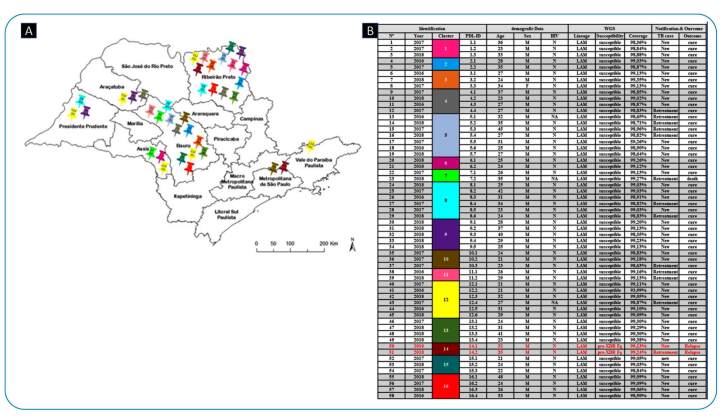
The location of the prisons where 58 clustered *M. tuberculosis* cases were identified in the state of São Paulo (A) and the information about the prison population (PP) and their isolates (B): year of diagnosis, cluster number, demographic information, lineage, drug-susceptibility pattern, TB case classification, and outcome**.** The clusters are color coordinated. **WGS:** whole-genome sequencing; **Pre XDR-Fq:** pre-extensively resistant to fluoroquinolone; **LAM:** Latin American-Mediterranean.

The average WGS depth of coverage for *M. tuberculosis* isolates was 84.5 (median 82.3). WGS analysis showed 76 non-clustered isolates, whereas the remaining 58 isolates were grouped into 16 clusters. The number of isolates within each cluster varied from two to seven (Figure 1). The isolates in each cluster varied by five or fewer SNPs, indicating closely related *M. tuberculosis* isolates and a recent transmission event^12^. 

Among the clustered isolates, 41 (70.6%) were classified as new TB cases, and 17 (29.4%) were from patients with a prior history of TB treatment. Cluster 1 (three isolates), cluster 2 (two isolates), cluster 3 (three isolates), cluster 13 (four isolates), and cluster 16 (four isolates) exclusively comprised new patients with TB. The other 11 clusters comprised 26 isolates from patients with both new and previously treated TB (Figure 1). 

**FIGURE 2:**
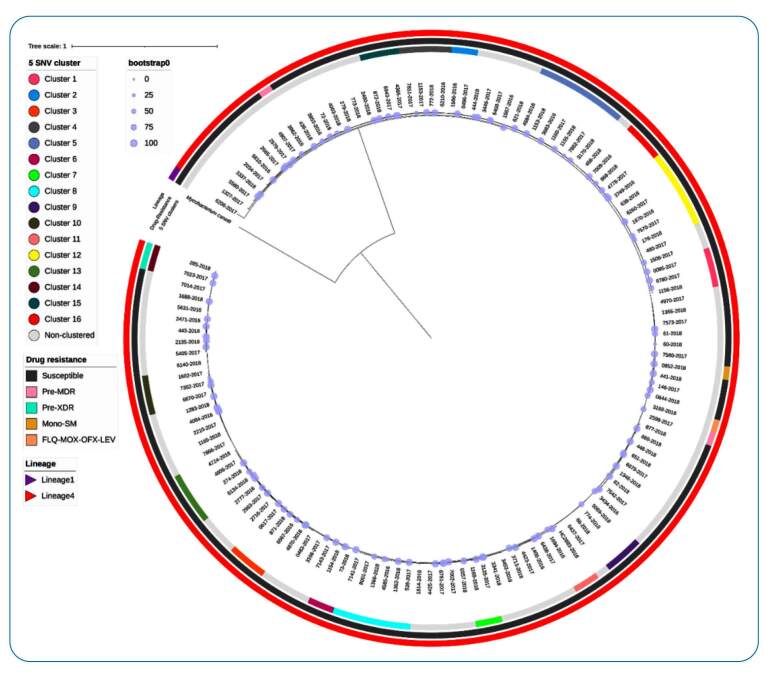
Phylogenetic tree showing the epidemiological relationship and drug susceptibility pattern of 134 *M. tuberculosis* lineages (left), and the 16 clustered (58 isolates) and 76 non-clustered isolates (right circle).

The two isolates in cluster 14 showed the same drug resistance pattern and were classified as pre-extensively drug-resistant (pre-XDR) with resistance to R, H, and Fq. The remaining four drug-resistant isolates were non-clustered (Figure 2).

The movement of prisoners from one prison to another was analyzed based on the identified clusters. Most clustered isolates were obtained from patients who moved through more than one prison during the study period. Eighteen out of 58 (31%) patients had overlapping stays in the same prison, and their isolates appeared in eight clusters (C1, C5, C6, C8, C9, C11, and C13). 

**FIGURE 3:**
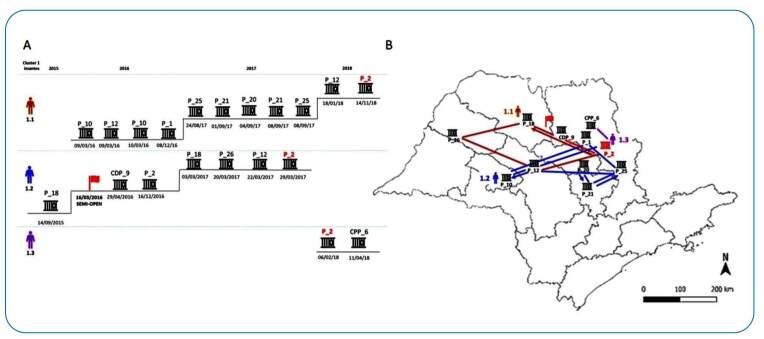
Different prisons (P) that the three patients with isolates from cluster 1 stayed in between 2016 and 2019.**(A)** Patient 1.2 was the first to be diagnosed in 2017, with the other two patients following in 2018. All three stayed in prison 2 (P2), shown in red. **(B)** Map depicts the moves of the patients of cluster 1 during the four-year period.

Studying the movement of the three patients with isolates in cluster 1 revealed that they stayed in the same prison for some time (Figure 3). Patient 1.2 was the first to be diagnosed in late 2017 in prison 2, and patients 1.1 and 1.3 were diagnosed with the same strain in February and November, 2018, respectively. A similar scenario was observed in other prisoners with clustered isolates. 

### Drug-resistant TB and lineages

Based on the WGS results, only six isolates (4.5%) showed resistance to one or more anti-TB drugs. Two isolates had mutations that conferred resistance to isoniazid (*katG* Ser315Thr). One deletion conferred streptomycin resistance (*gid* 351_351del). Another patient harbored a mutation that conferred resistance to fluoroquinolones (*gyrA* Ala90Val). Two isolates (Cluster 14) showed resistance to R (rpob_p.Ser450Leu), H (inhA_p.Ile21Thr), (katG_p.Gln127Pro), Fq (gyrA_p.Ser91Pro), (fabG1_c.-15C>T; pncA_p.Gly78Cys), (embB_p.Met306Ile), and (inhA_p.Ile21Thr fabG1_c.-15C). 

The results from the pDST were 100% concordant with WGS-identified mutations conferring resistance to R, H, E, and Fq. Of the three isolates for which pDST indicated resistance to pyrazinamide and ethionamide, only two had mutations known to confer resistance to these drugs. One isolate with a mutation conferring resistance to streptomycin, as identified by WGS, did not show any resistance in pDST.

The remaining 128 isolates without any known drug-resistance mutations were pan-susceptible based on pDST (100% concordance with WGS). The comprehensive phenotypic and genomic DST results are shown in Supplementary Table 1.

Only one isolate belonged to Lineage 1: East-African-India (EAI), while the remaining 133 isolates belonged to Lineage 4: Latin American-Mediterranean (LAM) (Figure 3). 

## DISCUSSION

There is an increasing consensus that the penitentiary system fuels the spread of TB within prisons and towards communities, driving the drug-resistant TB epidemic^20^.

In the last decade, although the number of individuals living in prison has increased by two-thirds globally, assessment of the prevalence of TB and DR-TB in the PP and surrounding communities remains challening^21^ ^-^ ^23^. In Brazil, the number of the PP from 2015 to 2020 increased by approximately 9,000 per year^7^. The vulnerability of the PP, a group that experiences a higher prevalence of HIV infection, addiction, low socioeconomic status, and malnutrition, in combination with the environmental factors of prison, such as insufficient ventilation and overcrowding, favors the transmission of TB and has resulted in several outbreaks, including DR-TB^24^ ^,^ ^25^. 

Our results show evidence of TB transmission within prisons in the northern region of São Paulo. The existence of the same strain, with fewer than five SNPs different between isolates, strongly indicates circulation and transmission within this enviroment^19^. 

A previous study conducted in a prison in Rio de Janeiro showed that most TB cases among prisoners were not due to reactivation of a previous infection or onset of disease due to a pre-existing latent infection, but rather a consequence of a recent, new infection by strains circulating in that penitentiary unit^26^.

Defining clusters as having five or fewer different SNPs, we grouped 58 *M. tuberculosis* isolates from the PP that had passed through 41 prisons in the northeast and northwest regions of the São Paulo state. Many stayed in the same prison for a short period. 

The frequent exchange of prisoners among prisons, as well as the movement from prisons to the community, creates gaps in TB prevention that put the community at risk^27^, as we observed and documented in this study.

However, prospective studies are needed to evaluate the impact of TB transmission among the PP and from this population to the community in Brazil, as indicated by our results, as well as other observational studies^6^ ^,^ ^28^.

The PP has complex and dynamic social interactions: an individual may enter multiple prisons, remain incarcerated for a long period of time, receive in-person visits from family, meet lawyers and legal representatives, and have frequent interactions with other prisoners and prison staff. Some prisoners are granted liberty and reintegrate into the community^29^ ^,^ ^30^. In Brazil, the PP might be granted temporary freedom on special occasions, such as holidays, during which they are allowed to go home and then return to the prison a few days later. 

The rise of DR-TB is a major challenge to the National Program for TB Control and is associated with inappropriate treatment of TB infection, non-adherence to treatment, and prison environments. Delayed detection of TB allows for continuous transmission inside the penitentiary system^6^ ^,^ ^31^. 

Although Central and South America have not yet been affected by extraordinarily high rates of drug-resistant TB in prisons, as seen in many Eastern European countries, the social and environmental conditions that drive TB and DR-TB transmission within prisons also drive the transmission of many other infectious diseases, including COVID-19^9^.

In this study, two isolates in cluster 14, which might have been transmitted within the penitentiary system, showed extensive drug resistance. However, the prevalence of drug-resistant TB in this study was much lower than that reported in previous studies on TB in prisons in other countries worldwide, mainly in Asia, Eastern Europe, and Africa^2^ ^,^ ^32^.

The low number of DR-TB cases in this study might be related to the Brazilian state regulation of therapeutic regimens for TB treatment, as standardization of drugs and dosage contributes to reducing the occurrence of drug resistance. The high cure rate for the studied group might be related to the directly observed treatment provided within the prisons. 

Consistent with previous epidemiological studies on TB in Brazil, most isolates in this study belonged to Lineage 4: LAM^18^.

This study had limitations because we could not sequence the isolates from all prisoners with TB diagnosed from 2016 to 2019, which were available in the Central Laboratory of *Mycobacteria*. Isolates did not grow or could not be sequenced, which may have restricted a more comprehensive analysis of TB transmission inside these prisons. An additional limitation was the inability to define the exact location of each prisoner within each prison during the study period. 

After this project was concluded, the research team presented the results to the São Paulo State Penitentiary Secretary staff and the Tuberculosis Control Program, suggesting a reduction in prisoner movements within and between prisons to reduce TB transmission. Additionally, movement of the prisoner should involve TB screening before and after using rapid molecular tests and isolating confirmed cases as soon as possible. WGS is a strong but expensive tool to monitor the dynamics of TB transmission within the penitentiary system in more detail.
