# Supplementary material for: New insights on tuberculosis transmission dynamics and drug susceptibility profiles among the prison population in Southern Brazil based on whole-genome sequencing
Source: Rev Soc Bras Med Trop. 2023 Feb 20;56:e0181-2022. doi: 10.1590/0037-8682-0181-2022 (PMC9957134; doi:10.1590/0037-8682-0181-2022)
Supplement: Supplementary file 1 [file 1678-9849-rsbmt-56-e0181-2022-supp1.pdf]

SUPPLEMENTARY TABLE 1: Information about all the isoles (clustered and non-clustered).

Identification				demografic Data			WGS			Notification & Ourcome	
Nº	Year	Cluster	PDL-ID	Age	Sex	HIV	Lineage	Susceptibility	Coverage	TB case	Outcome
1	2017	1	1.1	36	M	N	LAM	susceptible	98,36%	New	cure
2	2017		1.2	23	M	N	LAM	susceptible	98,84%	New	cure
3	2018		1.3	33	M	N	LAM	susceptible	98,88%	New	cure
4	2016	2	2.1	28	M	N	LAM	susceptible	99,03%	New	cure
5	2017		2.2	35	M	N	LAM	susceptible	98,87%	New	cure
6	2016	3	3.1	27	M	N	LAM	susceptible	99,13%	New	cure
7	2018		3.2	24	M	N	LAM	susceptible	99,35%	New	cure
8	2017		3.3	34	F	N	LAM	susceptible	99,13%	New	cure
9	2017	4	4.1	37	M	N	LAM	susceptible	98,85%	New	cure
10	2018		4.2	23	M	N	LAM	susceptible	99,02%	New	cure
11	2016		4.3	27	M	N	LAM	susceptible	98,87%	New	cure
12	2017		4.4	27	M	N	LAM	susceptible	98,83%	Retreatment	cure
13	2016	5	5.1	32	M	NA	LAM	susceptible	98,65%	Retreatment	cure
14	2018		5.2	35	M	N	LAM	susceptible	98,71%	Retreatment	cure
15	2017		5.3	45	M	N	LAM	susceptible	98,96%	Retreatment	cure
16	2018		5.4	27	M	N	LAM	susceptible	98,82%	Retreatment	cure
17	2017		5.5	31	M	N	LAM	susceptible	59,26%	New	cure
18	2016		5.6	25	M	N	LAM	susceptible	98,90%	New	cure
19	2018		5.7	27	M	N	LAM	susceptible	98,64%	New	cure
20	2018	6	6.1	25	M	N	LAM	susceptible	99,26%	New	cure
21	2018		6.2	24	M	N	LAM	susceptible	99,12%	New	cure
22	2017	7	7.1	26	M	N	LAM	susceptible	99,13%	New	cure
23	2018		7.2	35	M	NA	LAM	susceptible	99,27%	Retreatment	death
24	2018	8	8.1	25	M	N	LAM	susceptible	99,03%	New	cure
25	2017		8.2	42	M	N	LAM	susceptible	99,03%	New	cure
26	2016		8.3	31	M	N	LAM	susceptible	98,91%	New	cure
27	2017		8.4	34	M	N	LAM	susceptible	98,82%	Retreatment	cure
28	2017		8.5	23	M	N	LAM	susceptible	99,03%	New	cure
29	2018		8.6	24	M	N	LAM	susceptible	98,83%	Retreatment	cure
30	2018	9	9.1	28	M	N	LAM	susceptible	99,20%	New	cure
31	2018		9.2	37	M	N	LAM	susceptible	99,13%	New	cure
32	2018		9.3	40	M	N	LAM	susceptible	98,35%	New	cure
33	2018		9.4	29	M	N	LAM	susceptible	99,23%	New	cure
34	2018		9.5	25	M	N	LAM	susceptible	99,13%	New	cure
35	2017	10	10.1	24	M	N	LAM	susceptible	98,83%	New	cure
36	2017		10.2	21	M	N	LAM	susceptible	99,18%	New	cure
37	2017		10.3	23	M	N	LAM	susceptible	98,63%	Retreatment	cure
38	2016	11	11.1	26	M	N	LAM	susceptible	99,16%	Retreatment	cure

39	2018		11.2	29	M	N	LAM	susceptible	99,13%	Retreatment	cure
40	2017		12.1	21	M	N	LAM	susceptible	99,11%	New	cure
41	2016		12.2	21	M	N	LAM	susceptible	93,99%	New	cure
42	2018	12	12.3	32	M	N	LAM	susceptible	99,05%	New	cure
43	2017		12.4	27	M	NA	LAM	susceptible	98,87%	Retreatment	cure
44	2016		12.5	31	M	N	LAM	susceptible	99,10%	New	cure
45	2018		12.6	29	M	N	LAM	susceptible	99,09%	New	cure
46	2017		13.1	24	M	N	LAM	susceptible	99,30%	New	cure
47	2018	13	13.2	31	M	N	LAM	susceptible	99,29%	New	cure
48	2018		13.3	41	M	N	LAM	susceptible	99,30%	New	cure
49	2018		13.4	23	M	N	LAM	susceptible	99,38%	New	cure
50	2016	14	14.1	32	M	N	LAM	pre-XDR Fq	99,13%	New	Relapse
51	2018		14.2	35	M	N	LAM	pre-XDR Fq	99,24%	Retreatment	Relapse
52	2017		15.1	21	M	N	LAM	susceptible	99,05%	new	cure
53	2018	15	15.2	24	M	N	LAM	susceptible	99,03%	New	cure
54	2017		15.3	22	M	N	LAM	susceptible	98,84%	New	cure
55	2018		16.1	48	M	N	LAM	susceptible	99,09%	New	cure
56	2017	16	16.2	24	M	N	LAM	susceptible	99,09%	New	cure
57	2018		16.3	26	M	N	LAM	susceptible	99,06%	New	cure
58	2016		16.4	33	M	N	LAM	susceptible	98,50%	New	cure
59	2018		17	22	M	N	LAM	susceptible	99,12%	new	cure
60	2018		18	37	M	N	LAM	susceptible	99,25%	Retreatment	cure
61	2018		19	33	M	N	LAM	susceptible	99,19%	new	cure
62	2017	N	20	20	M	N	LAM	susceptible	99,11%	new	cure
63	2018	O	21	28	M	N	LAM	susceptible	98,62%	new	cure
64	2018	N	22	22	M	N	LAM	susceptible	98,74%	new	cure
65	2018		23	25	M	N	LAM	susceptible	99,03%	new	cure
66	2018	C	24	33	M	N	LAM	susceptible	99,25%	new	cure
67	2018	L	25	20	M	N	LAM	susceptible	99,20%	new	cure
68	2018	U	26	35	M	N	LAM	susceptible	98,95%	new	cure
69	2018	S	27	21	M	N	LAM	susceptible	99,17%	new	cure
70	2018	T	28	29	M	N	LAM	susceptible	99,09%	new	cure
71	2017	E	29	19	M	N	LAM	susceptible	99,13%	new	cure
72	2017	R	30	42	M	POS	LAM	susceptible	98,74%	Retreatment	cure
73	2018	E	31	30	M	N	LAM	susceptible	98,94%	Retreatment	Relapse
74	2018	D	32	43	M	N	LAM	susceptible	99,15%	new	cure
75	2018		33	31	M	N	LAM	susceptible	99,27%	new	cure
76	2018		34	23	M	N	LAM	susceptible	99,08%	new	cure
77	2018		35	29	F	N	LAM	Sm resistant	97,92%	new	cure
78	2018		36	41	M	NA	LAM	susceptible	99,30%	new	cure
79	2018		37	31	M	N	LAM	susceptible	99,32%	new	cure
80	2018		38	24	M	N	LAM	susceptible	99,21%	new	cure
81	2018		39	26	M	N	LAM	susceptible	99,09%	Retreatment	cure
82	2017		40	39	M	N	LAM	susceptible	99,14%	new	cure
83	2018		41	61	M	N	LAM	susceptible	99,18%	new	cure

84	2016		42	28	M	N	LAM	susceptible	99,00%	new	cure
85	2017		43	31	M	N	LAM	susceptible	99,00%	new	cure
86	2016		44	27	M	N	LAM	susceptible	98,78%	new	cure
87	2018		45	37	M	N	LAM	susceptible	99,19%	new	cure
88	2016		46	39	M	N	LAM	susceptible	99,21%	new	cure
89	2017		47	22	M	N	LAM	susceptible	99,01%	new	cure
90	2018		48	45	M	N	LAM	susceptible	99,21%	new	cure
91	2017		49	35	M	N	LAM	susceptible	99,21%	new	cure
92	2016		50	25	M	N	LAM	susceptible	99,28%	new	cure
93	2018		51	27	M	N	LAM	susceptible	99,10%	new	cure
94	2017		52	38	M	N	LAM	susceptible	99,03%	Retreatment	cure
95	2017		53	24	M	POS	LAM	H resistant	99,16%	Retreatment	cure
96	2017		54	35	M	N	LAM	susceptible	99,16%	Retreatment	cure
97	2017		55	33	M	N	LAM	susceptible	99,22%	new	cure
98	2016		56	43	M	N	LAM	susceptible	99,29%	Retreatment	cure
99	2017		57	20	M	N	LAM	susceptible	99,39%	Retreatment	cure
100	2017		58	33	M	N	LAM	susceptible	99,39%	Retreatment	Relapse
101	2018		59	24	F	N	LAM	Fq resistant	99,08%	new	cure
102	2018		60	34	M	N	LAM	susceptible	99,05%	new	cure
103	2018		61	42	M	N	LAM	susceptible	99,13%	new	cure
104	2016		62	44	M	N	LAM	susceptible	99,16%	Retreatment	cure
105	2017		63	32	M	N	LAM	susceptible	98,92%	new	cure
106	2016		64	61	M	N	LAM	susceptible	99,27%	new	cure
107	2018		65	38	M	N	LAM	susceptible	99,13%	new	cure
108	2016		66	33	M	N	LAM	susceptible	99,02%	new	cure
109	2016		67	37	M	N	LAM	H resistant	99,08%	Retreatment	Relapse
110	2018		68	38	M	N	LAM	susceptible	99,00%	new	cure
111	2016		69	43	M	N	LAM	susceptible	99,04%	new	cure
112	2017		70	31	M	N	LAM	susceptible	99,13%	new	cure
113	2017		71	41	M	N	LAM	susceptible	99,62%	new	cure
114	2018		72	36	M	N	LAM	susceptible	98,99%	new	cure
115	2017		73	43	M	N	LAM	susceptible	99,13%	Retreatment	cure
116	2017		74	28	M	N	LAM	susceptible	98,87%	new	cure
117	2017		75	35	M	POS	LAM	susceptible	99,19%	Retreatment	cure
118	2017		76	36	M	N	LAM	susceptible	99,06%	Retreatment	cure
119	2017		77	31	M	N	LAM	susceptible	98,96%	new	cure
120	2016		78	35	M	N	LAM	susceptible	99,12%	new	cure
121	2016		79	36	M	POS	LAM	susceptible	99,00%	Retreatment	cure
122	2017		80	36	M	N	LAM	susceptible	98,02%	new	cure
123	2018		81	24	M	N	LAM	susceptible	94,12%	new	cure
124	2017		82	35	M	N	EIA	susceptible	98,76%	new	cure
125	2017		83	23	M	N	LAM	susceptible	98,78%	new	cure
126	2017		84	26	M	N	LAM	susceptible	98,44%	new	cure
127	2017		85	55	M	N	LAM	susceptible	98,32%	new	cure
128	2017		86	24	M	N	LAM	susceptible	99,09%	new	cure

129	2017		87	27	M	N	LAM	susceptible	99,26%	new	cure
130	2017		88	20	M	N	LAM	susceptible	99,30%	new	Relapse
131	2017		89	28	M	N	LAM	susceptible	99,23%	new	cure
132	2017		90	33	M	N	LAM	susceptible	99,11%	Retreatment	cure
133	2017		91	36	M	N	LAM	susceptible	99,25%	Retreatment	cure
134	2017		92	26	M	N	LAM	susceptible	98,91%	Retreatment	cure

Legend: PDL-ID: identification number of each person deprived of liberty; WGS: whole genome sequencing; Sm- streptomycin; H: isoniazid; Fq: fluoquinole; Pre XDR-Fq: pre-extensively resistant to fluoquinolone; LAM: Latin American–Mediterranean; EIA: East-African-India.

References:

1. Abascal E, Herranz M, Acosta F, Agapito J, Cabibbe AM, Monteserin J, Ruiz Serrano MJ, Gijón P, Fernández-González F, Lozano N, Chiner-Oms Á, Cáceres T, Pintado PG, Acín E, Valencia E, Muñoz P, Comas I, Cirillo DM, Ritacco V, Gotuzzo E, García de Viedma D. Screening of inmates transferred to Spain reveals a Peruvian prison as a reservoir of persistent Mycobacterium tuberculosis MDR strains and mixed infections. Sci Rep. 2020 Feb 17;10(1):2704.
2. Adami AG, Gallo JF, Pinhata JM, Martins MC, Giampaglia CM and de Oliveira RS (2017) Modified protocol for drug susceptibility testing of MGIT cultures of Mycobacterium tuberculosis by the MGIT 960. Diagn Microbiol Infect Dis 87: 108-11.
3. Aldaoud N, Erashdi M, AlKhatib S, Abdo N, Al-Mohtaseb A and Graboski-Bauer A (2019) The utility of PAX8 and SATB2 immunohistochemical stains in distinguishing ovarian mucinous neoplasms from colonic and appendiceal mucinous neoplasm. BMC research notes 12: 1-6.
4. Andrews S 2015 FastQC: a quality control tool for high throughput sequence data. 2010 In.
5. Awofeso N (2010) Prisons as Social Determinants of Hepatitis C Virus and Tuberculosis Infections. Public Health Reports 125: 25-33.
6. Bolger AM, Lohse M and Usadel B (2014) Trimmomatic: a flexible trimmer for Illumina sequence data. Bioinformatics 30: 2114-20.
7. Broach S, Petrone M, Ryan J, Sivaram A and Gonsalves G (2019) Reservoirs of Injustice: How Incarceration for Drug-Related Offenses Fuels the Spread of Tuberculosis in Brazil. Global Health Justice Partnership Report, Yale Law School/Yale School of Public Health.
8. Sophie Broach, Jackson Institute for Global Affairs, 2019 Mary Petrone, Yale School of Public Health, PhD pre-candidate Juliet Ryan, MPH, Yale School of Public Health Anirudh Sivaram, Yale Law School, 2020

9. Coninx R, Maher D, Hernán R and Grzemska M (2000) Tuberculosis in prisons in countries with high prevalence. Bmj 320: 440-42.

10. Dara M, Grzemska M, Kimerling M, Reyes H and Zagorskiy A (2009) Guidelines for control of tuberculosis in prisons. Geneva: Tuberculosis Coalition for Technical Assistance. International Committee of the Red Cross.

11. Droznin, Maxwell et al. "Multidrug resistant tuberculosis in prisons located in former Soviet countries: A systematic review." PloS one vol. 12,3 e0174373. 23 Mar. 2017, doi:10.1371/journal.pone.0174373

12. Faksri K, Xia E, Tan JH, Teo Y-Y and Ong RT-H (2016) In silico region of difference (RD) analysis of Mycobacterium tuberculosis complex from sequence reads using RD-Analyzer. BMC genomics 17: 1-10.

13. Fazel S and Baillargeon J (2011) The health of prisoners. The Lancet 377: 956-65.

14. Gallo JF, Pinhata JMW, Saraceni CP and de Oliveira RS (2017) Evaluation of the BACTEC MGIT 960 system and the resazurin microtiter assay for susceptibility testing of Mycobacterium tuberculosis to second-line drugs. J Microbiol Methods 139: 168-71.

15. Gardy JL, Johnston JC, Sui SJH, Cook VJ, Shah L, Brodkin E, Rempel S, Moore R, Zhao Y and Holt R (2011) Whole-genome sequencing and social-network analysis of a tuberculosis outbreak. New England Journal of Medicine 364: 730-39.

16. Ginn S (2012) Prison environment and health. Bmj 345: e5921.

17. Gygli SM, Loiseau C, Jugheli L, Adamia N, Trauner A, Reinhard M, Ross A, Borrell S, Aspindzelashvili R, Maghradze N, et al. (2021) Prisons as ecological drivers of fitness-compensated multidrug-resistant Mycobacterium tuberculosis. Nature medicine 27: 1171-77.

18. Kamarulzaman A, Reid SE, Schwitters A, Wiessing L, El-Bassel N, Dolan K, Moazen B, Wirtz AL, Verster A and Altice FL (2016) Prevention of transmission of HIV, hepatitis B virus, hepatitis C virus, and tuberculosis in prisoners. The Lancet 388: 1115-26.

19. Letunic I and Bork P (2019) Interactive Tree Of Life (iTOL) v4: recent updates and new developments. Nucleic acids research 47: W256-W59.

20. Li H and Durbin R (2009) Fast and accurate short read alignment with Burrows–Wheeler transform. Bioinformatics 25: 1754-60.

21. Li H, Handsaker B, Wysoker A, Fennell T, Ruan J, Homer N, Marth G, Abecasis G and Durbin R (2009) The sequence alignment/map format and SAMtools. Bioinformatics 25: 2078-79.

22. Mabud TS, de Lourdes Delgado Alves M, Ko AI, Basu S, Walter KS, Cohen T, Mathema B, Colijn C, Lemos E, Croda J, Andrews JR. Correction: Evaluating strategies for control of tuberculosis in prisons and prevention of spillover into communities: An observational and modeling study from Brazil. PLoS Med. 2019 Mar 1;16(3):e1002764. doi: 10.1371/journal.pmed.1002764. eCollection 2019 Mar.PMID: 30822314

23. McKenna A, Hanna M, Banks E, Sivachenko A, Cibulskis K, Kernytsky A, Garimella K, Altshuler D, Gabriel S and Daly M (2010) The Genome Analysis Toolkit: a MapReduce framework for analyzing next-generation DNA sequencing data. Genome research 20: 1297-303.

24. McLaughlin S, Spradling P, Drociuk D, Ridzon R, Pozsik C and Onorato I (2003) Extensive transmission of Mycobacterium tuberculosis among congregated, HIV-infected prison inmates in South Carolina, United States. The international journal of tuberculosis and lung disease 7: 665-72.

25. Meiring C, Higgitt R, Dippenaar A, Roos E, Buss P, Hewlett J, Cooper D, Rogers P, de Klerk-Lorist LM and van Schalkwyk L (2020) Characterizing epidemiological and genotypic features of Mycobacterium bovis infection in wild dogs (Lycaon pictus). Transboundary and Emerging Diseases.

26. Olivia Cords, Leonardo Martinez, Joshua L Warren, Jamieson Michael O'Marr, Katharine S Walter, Ted Cohen, Jimmy Zheng, Albert I Ko 7, Julio Croda, Jason R Andrews. Incidence and prevalence of tuberculosis in incarcerated populations: a systematic review and meta-analysis. Lancet Public Health 2021 May;6(5):e300-e308.

27. Organization WH. 2011. *Guidelines for the programmatic management of drug-resistant tuberculosis-2011 update* (World Health Organization). (2020) Global tuberculosis report 2020: executive summary.

28. Phelan JE, O'Sullivan DM, Machado D, Ramos J, Oppong YE, Campino S, O'Grady J, McNerney R, Hibberd ML and Viveiros M (2019) Integrating informatics tools and portable sequencing technology for rapid detection of resistance to anti-tuberculous drugs. Genome medicine 11: 1-7.

29. Ponstingl H and Ning Z (2010) SMALT-a new mapper for DNA sequencing reads. F1000 Posters 1.

30. Sánchez A, Huber F, Massari V, Barreto A, Cesconi V, Saad M and Larouze B (2012) Extensive Mycobacterium tuberculosis circulation in a highly endemic prison and the need for urgent environmental interventions. Epidemiology & Infection 140: 1853-61.

31. Séraphin MN, Didelot X, Nolan DJ, May JR, Khan MSR, Murray ER, Salemi M, Morris Jr JG and Lauzardo M (2018) Genomic investigation of a Mycobacterium tuberculosis outbreak involving prison and community cases in Florida, United States. The American journal of tropical medicine and hygiene 99: 867.

32. Sidhu N, Clark M, Barba M, Armas-Cardona G, Zhang RJ, Tscherrig LS and Morgulis GEHF 2017 Tuberculosis, human rights and the law: a compendium of case law In.: University of Chicago Law School: International Human Rights Clinic.

33. Sosa L, Lobato M, Condren T, Williams M and Hadler J (2008) Outbreak of tuberculosis in a correctional facility: consequences of missed opportunities. The international journal of tuberculosis and lung disease 12: 689-91.

34. Tavoschi L, O'Moore É and Hedrich D (2019) Challenges and opportunities for the management of infectious diseases in Europes' prisons: evidence-based guidance. The Lancet Infectious Diseases 19: e253-e58.

35. Valim ARDM, Possuelo LG, Cafrune PI, Borges M, Ribeiro MO, Rossetti MLR and Zaha A (2006) Evaluation and genotyping of multidrug-resistant cases of tuberculosis in southern Brazil. Microbial Drug Resistance 12: 186-91.

36. Walker TM, Ip CL, Harrell RH, Evans JT, Kapatai G, Dedicoat MJ, Eyre DW, Wilson DJ, Hawkey PM, Crook DW, et al. (2013) Whole-genome sequencing to delineate Mycobacterium tuberculosis outbreaks: a retrospective observational study. Lancet Infect Dis 13: 137-46.

37. Walter KS, Martinez L, Arakaki-Sanchez D, Sequera VG, Sanabria GE, Cohen T, Ko AI, García-Basteiro AL, Rueda ZV and López-Olarte RA (2021) The escalating tuberculosis crisis in central and South American prisons. The Lancet.

38. Warren JL, Grandjean L, Moore DA, Lithgow A, Coronel J, Sheen P, Zelner JL, Andrews JR and Cohen T (2018) Investigating spillover of multidrug-resistant tuberculosis from a prison: a spatial and molecular epidemiological analysis. BMC medicine 16: 1-9.

39. Witbooi P and Vyambwera SM (2017) A model of population dynamics of TB in a prison system and application to South Africa. BMC research notes 10: 1-8.

40. Xia E, Teo Y-Y and Ong RT-H (2016) SpoTyping: fast and accurate in silico Mycobacterium spoligotyping from sequence reads. Genome medicine 8: 1-9.
